# Impact of Climate Change on the Potential Distribution of *Belamcanda chinensis* Under Multiple Climatic Scenarios

**DOI:** 10.1002/ece3.72414

**Published:** 2025-10-30

**Authors:** Wei Lin, Feiran Hu, Guochun Fan, Qingxia Zhang, Min Deng, Xiangxin Xu, Yibing Liu, Junsheng Qi

**Affiliations:** ^1^ College of Biology and Food Engineering Chongqing Three Gorges University Chongqing China; ^2^ College of Environmental and Chemical Engineering Chongqing Three Gorges University Chongqing China

**Keywords:** *Belamcanda chinensis* (L.) Redouté, ecological niche theory, environmental adaptability, MaxEnt

## Abstract

*Belamcanda chinensis* (L.) Redouté, a perennial herb in the Iridaceae family, exhibits a broad spectrum of medicinal properties. Identifying the optimal habitat for *B. chinensis* is crucial for establishing a scientific basis for the conservation of its genetic and ecological resources. In our study, the MaxEnt model was utilized to predict the potential distribution of *B. chinensis* under multiple climatic scenarios, while exploring potential ecological niche shifts through the lens of ecological niche theory. The results revealed that *B. chinensis* was primarily distributed across subtropical and temperate regions of China, with a notably extensive distribution range. Projections under all future climate scenarios suggested an expansion of suitable habitats for *B. chinensis* . Temperature, slope, and precipitation were identified as the primary environmental factors influencing its distribution. Furthermore, although future projections indicated a reduction in shared resources between shoots and dry habitats, the availability of usable resources was expected to increase, thereby enhancing the plant's environmental adaptability. Our findings could provide essential insights for the conservation, sustainable use, and management of *B. chinensis* resources.

## Graphical abstract

Projections under all future climate scenarios suggested an expansion of suitable habitats for *B. chinensis* . Furthermore, although future projections indicated a reduction in shared resources between shoots and dry habitats, the availability of usable resources was expected to increase, thereby enhancing the plant's environmental adaptability.

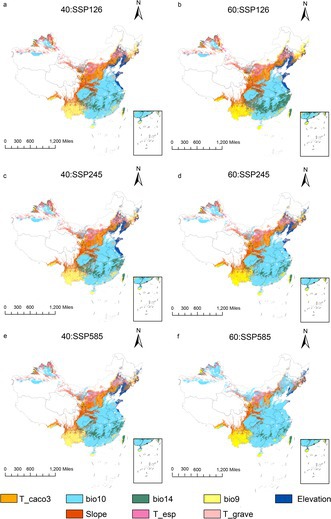

## 1 Introduction

The study of global climate change has long been a critical area of concern, with its profound impact on biodiversity loss emerging as one of the most pressing challenges facing humanity. Human activities, compounded by climate change, are accelerating environmental degradation, resulting in significant threats to biodiversity that jeopardize ecosystem stability and the services they provide. This issue represents a major global challenge, requiring urgent attention and integrated strategies for mitigation and adaptation (Mantyka‐pringle et al. 2012). The impact of climate change on species distribution has emerged as a critical area of research, with significant scholarly attention devoted to the shifts in spatial patterns of species globally (Jump and Peñuelas 2005). Furthermore, in the context of global warming, which has driven significant shifts in the geographic distribution of numerous species (Bellard et al. 2013), future climate change is expected to accelerate the migration of plant species toward higher latitudes. Such alterations in species distributions will profoundly affect how climate variability manifests across landscapes, as surface vegetation plays a critical role in shaping atmospheric properties (Thuiller et al. 2005; Fitzpatrick et al. 2008). Under this trend, the entire ecosystem will face severe threats (Bosch‐Belmar et al. 2021), with numerous species experiencing habitat shifts, range contractions, or even complete loss, ultimately leading to a decline in global biodiversity. A comprehensive understanding of habitat characteristics and species' geospatial distributions is therefore essential for biodiversity conservation planning and the prediction of suitable habitat areas. Such knowledge forms the foundation for identifying the key drivers of ecological habitat dynamics and species diversity evolution across various spatial scales (Elith et al. 2011). As such, the investigation of species suitability zones is of critical importance in addressing these challenges.

The continuous advancement of geographic information systems (GIS) and the development of innovative statistical techniques to analyze GIS data have significantly contributed to the proliferation of ecological niche modeling. Among these models, the Genetic Algorithm for Rule Set Prediction (GARP), Domain Distance, Generalized Linear Model (GLM), Random Forest (RF), Maximum Entropy (MaxEnt), and Generalized Additive Model (GAM) are particularly prominent (Sun et al. 2020; Li, Liu, et al. 2022). The MaxEnt model, grounded in the theory of maximum entropy, estimates the future distribution of a species by iteratively sampling from a large number of distributions and selecting the one that has the highest entropy (i.e., spread) based on the current distribution of the species as well as other parameter constraints (Warren and Seifert 2011; Jiang 2019). Where the stochastic nature of a species distribution is modeled to maximize its spread using current data. This process involves converting the problem into a probabilistic model, where the stochastic nature of species distributions is expressed as a probability distribution. The solution is represented by the optimal probability distribution (Yingjie and Guangliang 1996). As additional environmental variables are incorporated and iterations during the L‐BFGS‐B (Limited‐memory Broyden–Fletcher–Goldfarb–Shanno algorithm with Boundary constraints) optimization increase, the entropy value progressively rises until a state of maximum entropy is achieved, providing the most accurate representation of the species' potential distribution (Xu 2019). Among various SDMs, MaxEnt has gained prominence in recent years due to its superior predictive performance and geographic consistency (Phillips and Dudík 2008; Liu et al. 2019; Shabani et al. 2019). Its widespread adoption is also attributed to its effectiveness in scenarios with limited sample sizes, ease of use, and relatively short runtime (Pearson et al. 2007; Jha and Jha 2021). Currently, SDMs, including MaxEnt, are widely applied in forecasting the potential distributions of invasive species, as well as identifying suitable habitats for endangered and medicinal plants (Padalia et al. 2014; Wang et al. 2023, 2024; Zou et al. 2023). These applications underscore the growing importance of SDMs in biodiversity conservation and ecological research.

The concept of the “niche” was first introduced by Grinnell in 1917 (Grinnell 1917). However, its precise definition and interpretation remain topics of significant debate among researchers (Niu et al. 2009; Peng and Wang 2016). Classical ecological niche theory posits that differences in the utilization of environmental resources among species are one of the fundamental mechanisms facilitating species coexistence (Chesson 2000). Two quantitative metrics frequently employed in ecological niche studies are ecological niche breadth and ecological niche overlap. Ecological niche breadth refers to the range of environmental resources a species utilizes, which serves as an indicator of the species' ecological adaptability and distribution magnitude (Smith 1982; Carscadden et al. 2020). By applying ecological niche theory, the quantitative analysis of a species' niche across different temporal and spatial scenarios provides a deeper understanding of its ecological niche characteristics.

*Belamcanda chinensis* is a perennial herb of the Iridaceae family, valued for its rhizome, which has long been utilized as a medicinal agent with properties that promote the clearance of heat and toxins, the elimination of phlegm, and the induction of diaphoresis (Chinese Pharmacopoeia Commission 2020). With a history of over two millennia in traditional Chinese medicine (TCM), it has often been employed as a principal ingredient in treatments for bronchial asthma (Liu, Zhu, et al. 2022). Modern pharmacological studies have demonstrated its diverse bioactive properties, including anti‐inflammatory, antibacterial, antiviral, antitumor, and neuroprotective effects (Guan et al. 2015; Yin 2020). Over 40 isoflavonoid monomers have been isolated from *B. chinensis* (Liu, Zhu, et al. 2022), and these compounds have been widely used in clinical applications. Their therapeutic efficacy is highly regarded within both domestic and international Chinese medicine communities (Liu, Chen, and Xiong 2003). In addition to its medicinal value, *B. chinensis* is a dual‐purpose plant with striking orange‐red flowers, contributing to its ornamental appeal. In recent years, growing demand for *B. chinensis* has led to rising market prices, while wild populations have experienced a significant decline. However, research on the ecological distribution of *B. chinensis* and the potential shifts in its habitats under future climate change scenarios remains limited. Considering the profound implications of climate change on plant distribution, investigating the potential distribution of *B. chinensis* under future climate scenarios is crucial for resource conservation and the sustainable development of medicinal applications.

A broader analysis of the ecological requirements for Chinese medicinal plants is needed, so that the strategic planning of where to produce them on an agricultural scale can be integrated with future climate change scenarios and assessment of other factors such as the socio‐economic conditions. Our study aimed to achieve three objectives: (1) to predict the potentially suitable areas for *B. chinensis* japonica and assess their dynamics under various climatic scenarios; (2) to identify the primary environmental factors influencing the potentially suitable areas of *B. chinensis* japonica; (3) to explore the ecological niche characteristics of *B. chinensis* japonica under different climatic scenarios. We hope that our findings will provide a robust scientific basis for the conservation and sustainable development of *B. chinensis* resources.

## 2 Data Sources and Research Methods

### 2.1 Data Sources and Processing

#### 2.1.1 Data and Distribution Point Processing of *B. chinensis* With Curved Stem

**FIGURE 1:**
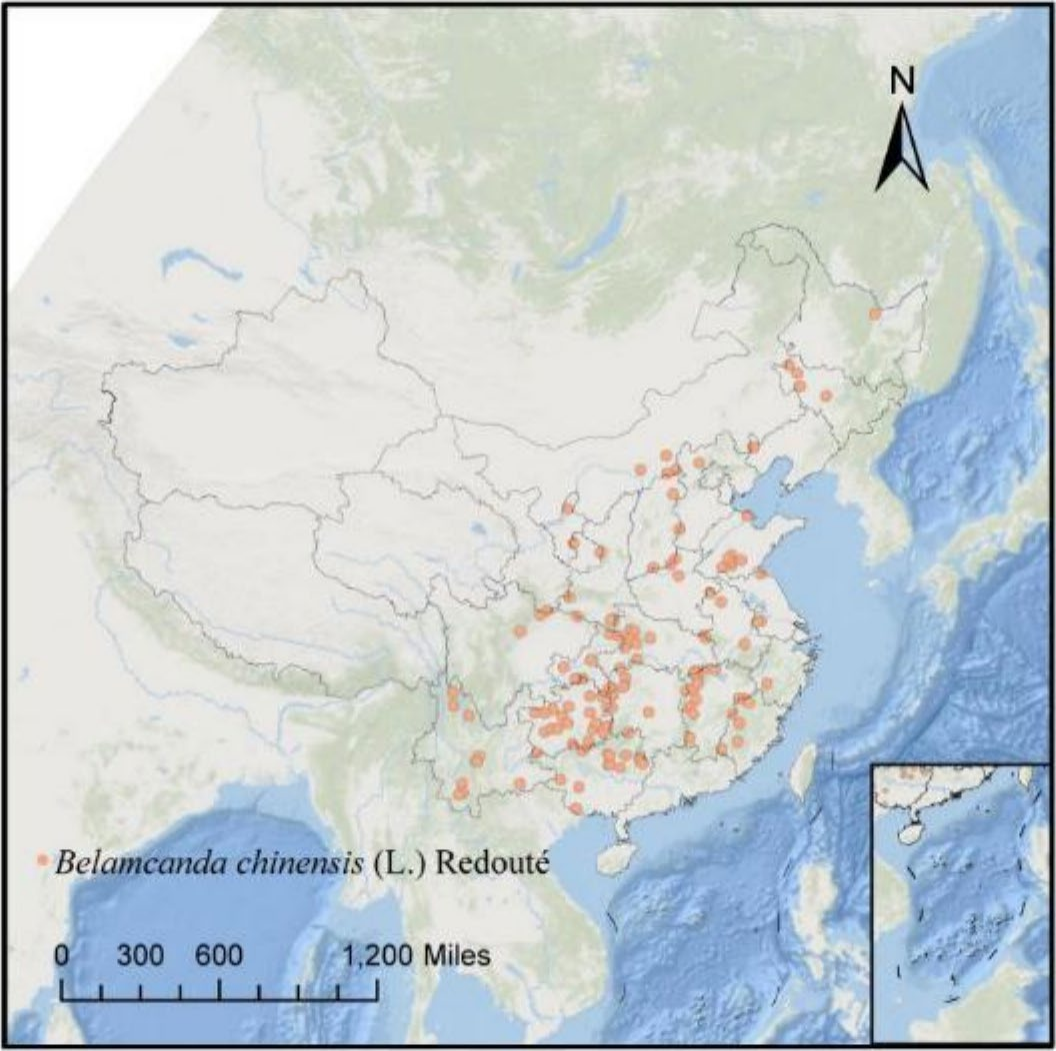
Geographical distribution of *B. chinensis*.

The geographic distribution data for *B. chinensis* were obtained from the China Virtual Herbarium (https://www.cvh.ac.cn/index.php) and the Global Biodiversity Information Facility (GBIF, https://www.gbif.org/). The initial distribution points of *B. chinensis* were obtained from occurrence records within China, encompassing both natural habitats and cultivated areas, with particular focus on post‐1980 collections. Using these records, the distribution points of Sagittaria in China were selected for analysis. To ensure data quality, the ENMtools package in R software was employed to filter out erroneous and duplicated entries. Following this data cleaning process, a total of 138 valid geographic distribution points for Sagittaria were retained (Figure 1).

#### 2.1.2 Environmental Variable Data Sources and Preprocessing

In our study, 35 environmental variables were selected, with climate data sourced from the WorldClim database (https://worldclim.org). The bioclimatic factors include both current (1970–2000) and future (2041–2060, 2061–2080) climate data. A set of 19 commonly used bioclimatic variables, labeled bio1 through bio19, was included in the analysis. Current climate data cover the period from 1970 to 2000, with a spatial resolution of 2.5 arcseconds (approximately 5 km). Future climate projections were obtained from the BCC‐CSM2‐MR model, as part of the Coupled Model Intercomparison Project Phase 6 (CMIP6) (Yang et al. 2023), using three representative socio‐economic pathways (SSPs): the green growth pathway (ssp126), the moderate growth pathway (ssp245), and the high growth pathway (ssp585) (Li et al. 2023). The core distinction among these SSP pathways lies in their divergent socioeconomic challenges and projected radiative forcing levels; SSP126 aims to limit warming to below 2°C, SSP245 leads to intermediate warming (approximately 2°C–3°C), while SSP585 may result in catastrophic warming exceeding 4°C. Soil type data were acquired from the Harmonized World Soil Database (https://www.fao.org/soils‐portal/soil‐survey/soil‐maps‐anddatabases/harmonized‐world‐soil‐databasev12/en/), developed by the Food and Agriculture Organization of the United Nations (FAO) and the International Institute for Applied Systems Analysis (IIASA). The database primarily utilizes the FAO‐90 soil classification system, with data retrieved from the Geospatial Data Cloud (http://www.gscloud.cn/) at a spatial resolution of 90 m.

**FIGURE 2:**
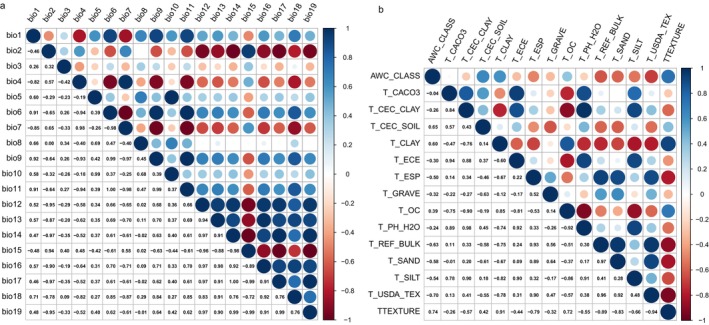
Spearman correlation analysis between bioclimatic factors (a) and soil factors (b).

Given the potential for strong spatial correlations between variables within groups of bioclimatic and soil factors, we conducted a correlation analysis for each group to mitigate the risk of model overfitting due to multicollinearity (Sillero 2011) (Figure 2). Based on the results of the correlation analysis and the jackknife test, factors with a contribution of less than 1% were manually excluded. For highly correlated variables (correlation coefficient > 0.8), only the most relevant variable for model interpretation was retained for further analysis (Radosavljevic and Anderson 2014). Ultimately, eight environmental factors were selected for the final model: three bioclimatic factors, three soil factors, and two topographic factors (Table 1).

**TABLE 1:** Environmental factors included in modeling.

No.	Indicators	Abbreviation	Description	Source	Included
1	Bioclimate	Bio1	Annual mean temperature	WorldClim	
2		Bio2	Mean diurnal temperature range (mean of monthly [max temp–min temp])		
3		Bio3	Isothermality (BIO2/BIO7) (×100)		
4		Bio4	Temperature seasonality		
5		Bio5	Max temperature of warmest month		
6		Bio6	Min temperature of coldest month		
7		Bio7	Temperature annual range (BIO5–BIO6)		
8		Bio8	Mean temperature of wettest quarter		
9		Bio9	Mean temperature of driest quarter		✔
10		Bio10	Mean temperature of warmest quarter		✔
11		Bio11	Mean temperature of coldest quarter		
12		Bio12	Annual precipitation		
13		Bio13	Precipitation of wettest month		
14		Bio14	Precipitation of driest month		✔
15		Bio15	Precipitation seasonality		
16		Bio16	Precipitation of wettest quarter		
17		Bio17	Precipitation of driest quarter		
18		Bio18	Precipitation of warmest quarter		
19		Bio19	Precipitation of coldest quarter		
20	Soil	AWC_CLASS	Available water capacity class	HWSD	
21		T_GRAVEL	Topsoil Gravel content		✔
22		T_SAND	Topsoil sand fraction		
23		T_CLAY	Topsoil clay fraction		
24		T_USDA_TEX	Topsoil USDA texture classification		
25		T_REF_BULK	Topsoil reference bulk density		
26		T_OC	Topsoil organic carbon		
27		T_PH_H2O	Topsoil pH (H_2_O)		
28		T_CEC_CLAY	Topsoil cation exchange capacity of the clay fraction		
29		T_CEC_SOIL	Topsoil cation exchange capacity		
30		T_CACO3	Topsoil calcium carbonate		✔
31		T_ESP	Topsoil exchangeable sodium percentage		✔
32		T_ECE	Topsoil electrical conductivity		
33		T_SILT	Topsoil silt content		
34	Topographic	Elevation	Elevation	SRTM	✔
35		SLOPE	SLOPE		✔

### 2.2 Establishment and Optimization of MaxEnt Model

The MaxEnt model was used to predict suitable habitats for *B. chinensis* , incorporating species occurrence data and environmental variables. The model parameters were set as follows: 75% of the species occurrence data were randomly selected for the training dataset, while the remaining 25% served as the testing dataset. The model was run with 10 repetitions, each consisting of a maximum of 10,000 iterations (Yuan et al. 2021). Model accuracy was assessed using the area under the receiver operating characteristic curve (AUC). The AUC is a commonly used metric for evaluating prediction accuracy, with values ranging from 0 to 1. An AUC value of 1 indicates perfect accuracy, while values below 0.7 indicate poor performance, values between 0.7 and 0.8 suggest moderate accuracy, and values above 0.9 reflect excellent predictive performance (Phillips et al. 2006). To assess the influence of each environmental variable, the jackknife test option in MaxEnt was applied (Pearson et al. 2007; Shcheglovitova and Anderson 2013), allowing the relative importance of each variable in model development to be determined.

The MaxEnt model was further optimized using the ENMeval package in R, with adjustments made to the regularization multiplier (RM) and feature combinations (FC) (Phillips and Dudík 2008). The RM had 11 possible values, ranging from 0.5 to 5, with an interval of 0.5. The feature combinations included five types: linear (L), quadratic (Q), hinge (H), product (P), and threshold (T) (Bohl et al. 2019). Six feature combinations were tested: L, LQ, H, LQH, LQHP, and LQHPT, resulting in a total of 66 possible combinations. The combination with the lowest AICc value (typically, delta.AICc = 0) was selected for final MaxEnt modeling.

### 2.3 Suitability Area Analysis

The results from MaxEnt were imported into ArcGIS 10.2 for format conversion and reclassification (Jiang et al. 2022). The natural breaks classification method was used to categorize the suitability of *B. chinensis* habitats into four classes: unsuitable areas (0–0.1059), low suitability areas (0.1059–0.3255), moderate suitability areas (0.3255–0.6392), and high suitability areas (0.6392–1). The centroid change tool, available through the SDMToolbox v2.5 ArcGIS plugin, was employed to visualize changes in area and centroid displacement within the suitability regions under both current and future climate scenarios (Yan et al. 2022; Wang et al. 2023).

### 2.4 Multivariate Environmental Similarity Surface (MESS) and Most Dissimilar Variables (MoD) Analysis

The Multivariate Environmental Similarity Surface (MESS) is a tool used to assess the degree of similarity (S) between current climatic scenarios and those projected under different future climate scenarios for a species. The MESS was employed to analyze the potential suitability of regions under various future climate scenarios. The current climate bioclimatic variables for *B. chinensis* were used as the reference layer. The primary drivers of change were identified by analyzing the most dissimilar variables. In regions where *B. chinensis* is predicted to occur, MESS was applied to evaluate the similarity between current climate scenarios and future climate scenarios (Wang et al. 2024). The maximum value of S is 100, which indicates that the future climate at a given location is identical to the reference layer, meaning the climate is normal. When 0 ≤ S < 100, smaller values of S indicate greater environmental divergence. A value of S < 0 signifies that one or more bioclimatic variables fall outside their corresponding ranges, marking the point as a climate anomaly. The variable exhibiting the greatest deviation from the multivariate environmental similarity surface is considered the most dissimilar and may be a key factor influencing species' geographic migration. This result was obtained using the density.tool.novel function for MaxEnt models (Elith et al. 2010; Li et al. 2016).

### 2.5 Ecological Niche Analysis

A comparative analysis of the ecological niche characteristics of *B. chinensis* was conducted between current conditions and future climate scenarios. Ecological width was assessed using ENMTools software, while the degree of ecological niche overlap was analyzed and visualized with the “ecospat” package in R (Di Cola et al. 2017). The degree of overlap between two ecotopes was quantified using Schoener's D coefficient, which ranges from 0 (indicating no overlap) to 1 (indicating complete overlap). This coefficient reflects the extent of environmental and geographic similarity between the two ecotopes in question (Schoener 1970; Fourcade et al. 2014).

The ecological overlap index is calculated as (Warren et al. 2010):

$$D\left(Px,Py\right)=1-\frac{1}{2}\sum_{i}|P_{\mathrm{xi}}-P_{\mathrm{yi}}|$$

where *D*(*P*_x_, *P*_y_) represents the niche overlap between species X and species Y; $P_{\mathrm{xi}}$ and $P_{\mathrm{yi}}$ denote the number of individuals of species X and species Y utilizing resource i (i = 1, 2, …, *n*), respectively.

The ecological niche breadth is calculated using the Levins model:

$$B_{i}=-\sum_{j=1}^{R}P_{\mathrm{ij}}\cdot \log P_{\mathrm{ij}}$$

where *B*_*i*_ is the Levins niche breadth of species *i*; $P_{\mathrm{ij}}$ represents the frequency of species *i* utilizing resource *j* relative to all resources utilized; and *R* denotes the number of available resource categories in the environment.

## 3 Result

### 3.1 MaxEnt Model Optimization Results

**FIGURE 3:**
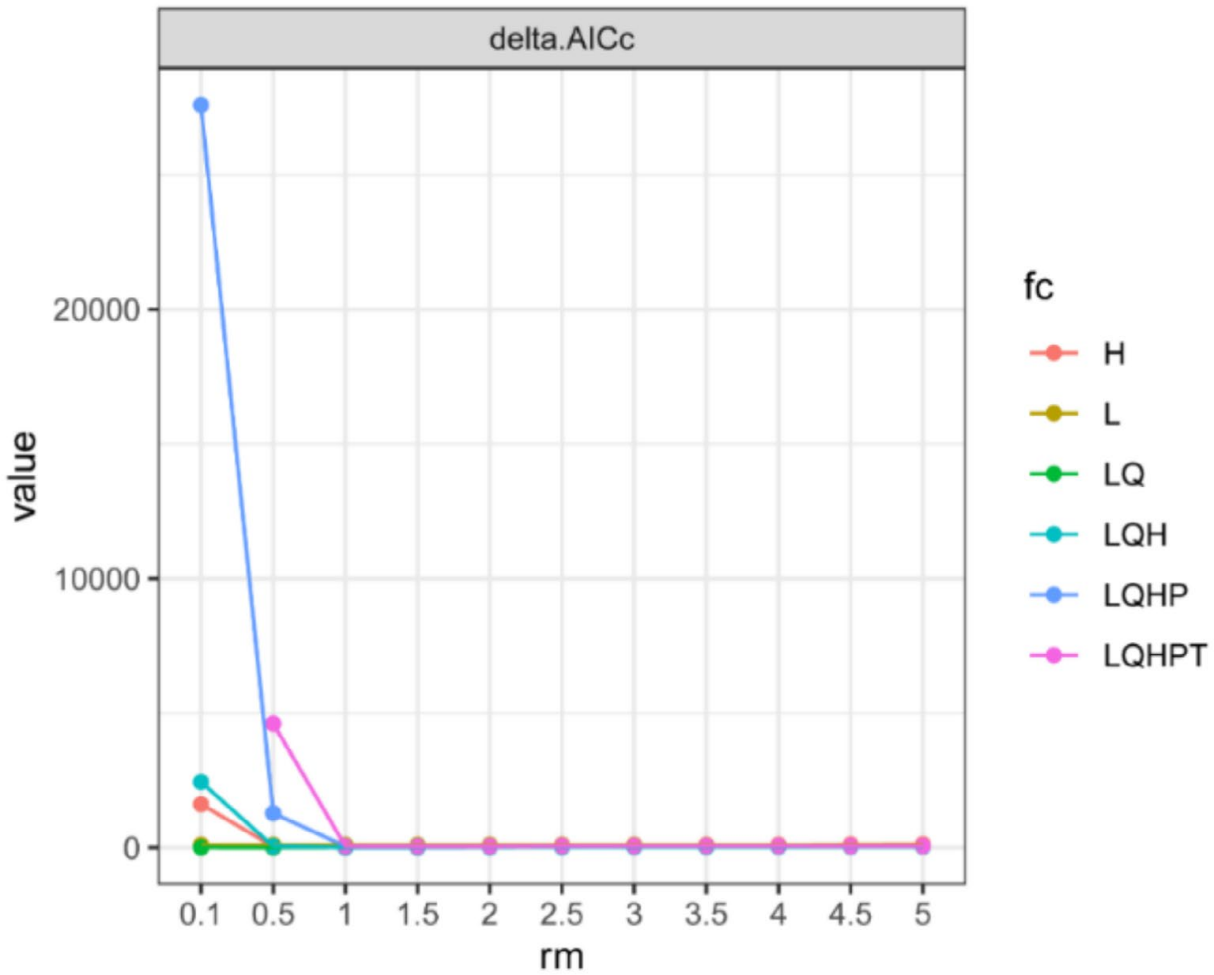
MaxEnt model parameter combinations (H, Hinge; L, Linear; LQ, Linear + Quadratic; LQH, Linear + Quadratic + Hinge; LQHP, Linear + Quadratic + Hinge + Product; LQHPT, Linear + Quadratic + Hinge + Product + Threshold).

**FIGURE 4:**
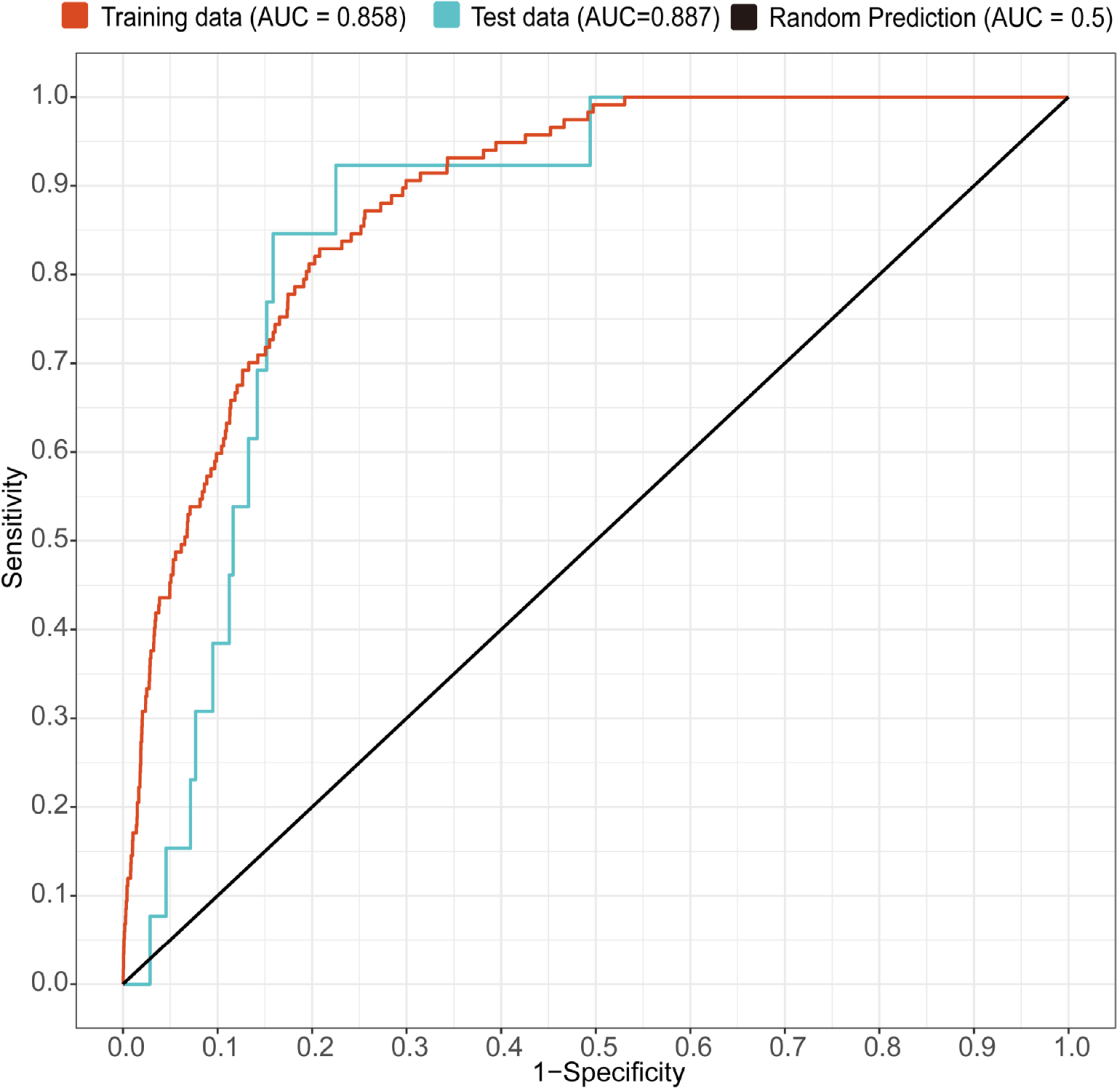
ROC curve for model evaluation in MaxEnt.

When using the default parameters of the MaxEnt model to predict the suitable habitat of *B. chinensis* , the AUC value for the training data was 0.856. After optimizing the MaxEnt model parameters using the ENMeval package in R (Figure 3), the optimal parameter combination for predicting the suitable habitat of *B. chinensis* in China was identified as FC = LQH and RM = 1. Under this parameter combination, the delta AICc was 0, indicating a lower value compared to the default settings. The AUC value for the training data with these optimal parameters increased to 0.887 (Figure 4), demonstrating improved prediction accuracy.

### 3.2 Suitable Areas for *B. chinensis* Under Current Climate Scenarios

**FIGURE 5:**
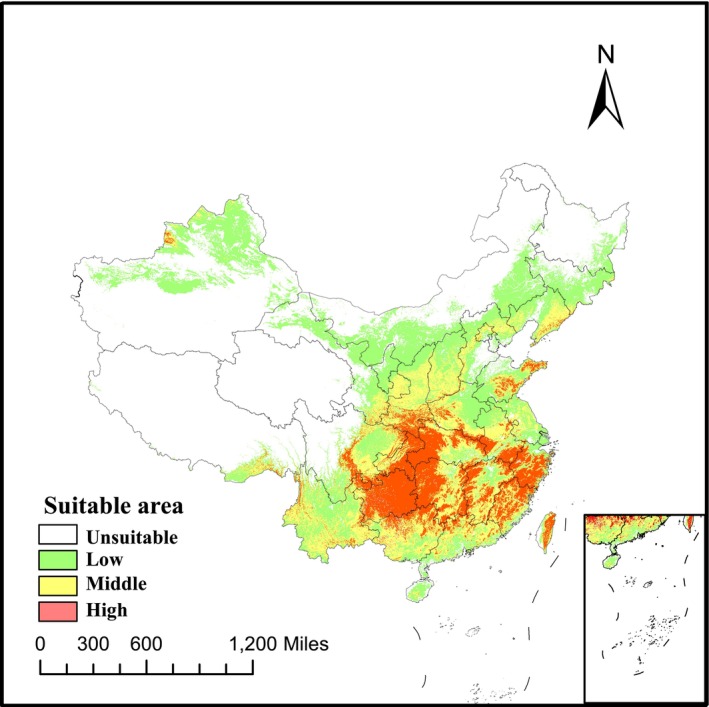
Suitable area of *B. chinensis* under current climate scenarios.

Using ArcGIS 10.2, we mapped the predicted suitable habitat of *B. chinensis* under current climatic scenarios and calculated the proportion of each suitability class (Figure 5). The distribution of *B. chinensis* was primarily concentrated in the subtropical and temperate regions of China, including the provinces of Fujian, Zhejiang, Guangdong, Guangxi, Shaanxi, Sichuan, Guizhou, Hubei, Henan, and Yunnan. Based on the prevailing climatic scenarios, the total area of suitable habitat for *B. chinensis* was estimated to be approximately 435.5 × 10^4^ km^2^, representing around 43.55% of China's total land area. The area of highly suitable habitat was approximately 86.29 × 10^4^ km^2^, which constituted about 9.21% of China's total land area, indicating that a significant portion of the suitable habitat was highly favorable for the species. The regions with the highest suitability were primarily located in Guizhou, Chongqing, Taiwan, Fujian, and Jiangsu, while areas with moderate suitability were mainly found in Guangxi, Shaanxi, Hebei, and Sichuan. Regions with low suitability were concentrated in Yunnan, Inner Mongolia, and Jilin. The MaxEnt model's predictions were generally consistent with the actual geographic distribution of *B. chinensis* in China, confirming the reliability of the model's projections.

### 3.3 Regional Distribution of Suitability for *B. chinensis* Under Future Climate Scenarios

**FIGURE 6:**
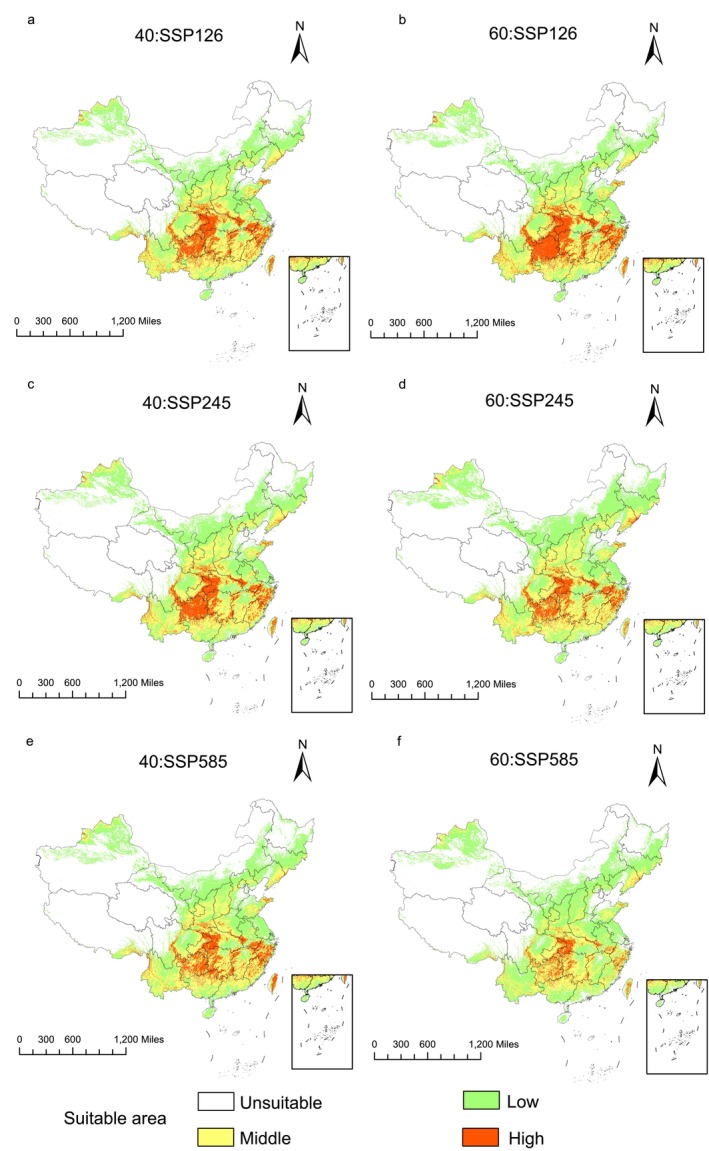
Suitable distribution areas of *B. chinensis* in SSP126 (a, b), SSP245 (c, d), SSP 585 (e, f) scenarios in the 2041–2060s (a, c, e) and 2061–2080s (b, d, f).

In comparison to the prevailing climatic scenarios, the prospective climate scenarios indicated the following trends (Figure 6; Table 2): under the SSP126 scenario, the area of suitable habitat exhibited a declining trend, whereas under the SSP245 scenario, the habitat area showed an expansion. In contrast, the SSP585 scenario indicated an increase in the suitable habitat area from 2041 to 2060, followed by a decline from 2061 to 2080. Overall, the change in the area of suitable habitat for *B. chinensis* in response to future climate scenarios was relatively modest, with area changes ranging from 4.27 × 10^4^ to 40.65 × 10^4^ km^2^, corresponding to a variation of 0.98%–9.33%.

**TABLE 2:** Suitable area under different climatic scenarios (×10^4^ km^2^).

Period	Climate scenario	Low‐suitability area	Moderately suitable area	Highly suitable area	Total suitable area
Curren		217.9	131.35	86.3	435.55
2041–2060	SSP126	215.01	153.61	62.64	431.26
	SSP245	234.42	155.05	59.98	449.45
	SSP585	242.79	152.7	52.92	448.41
2061–2080	SSP126	203.89	140.92	76.29	421.1
	SSP245	258.38	166.97	50.83	476.18
	SSP585	265.29	131.03	28.06	424.38

*Note:* The green growth pathway (SSP126), the moderate growth pathway (SSP 245), and the high growth pathway (SSP585).

In comparison to the prevailing climatic scenarios, regions exhibiting low suitability were predicted to expand under all future climate scenarios, except SSP126. Medium‐suitability areas showed an increasing trend in all scenarios, except for SSP126 during the 2061–2080 period. On the other hand, high‐suitability areas declined in area across all future climate scenarios because the regions most susceptible to global warming were those with high suitability. In the SSP585 scenario for the period 2061–2080, the area of highly suitable habitat was predicted to undergo the most significant decline, with a projected reduction of 67.49%. The regions exhibiting an increase in low‐suitability areas were primarily located in Xinjiang, Inner Mongolia, and Jilin, while those with an expansion of medium‐suitability areas were concentrated in Yunnan, Fujian, and Guangxi. The regions showing a decline in high‐suitability areas were predominantly situated in Guizhou, Hubei, and Chongqing.

### 3.4 Dynamic Changes in the Suitability Distribution Area of *B. chinensis* Under Different Climatic Scenarios and Periods

**FIGURE 7:**
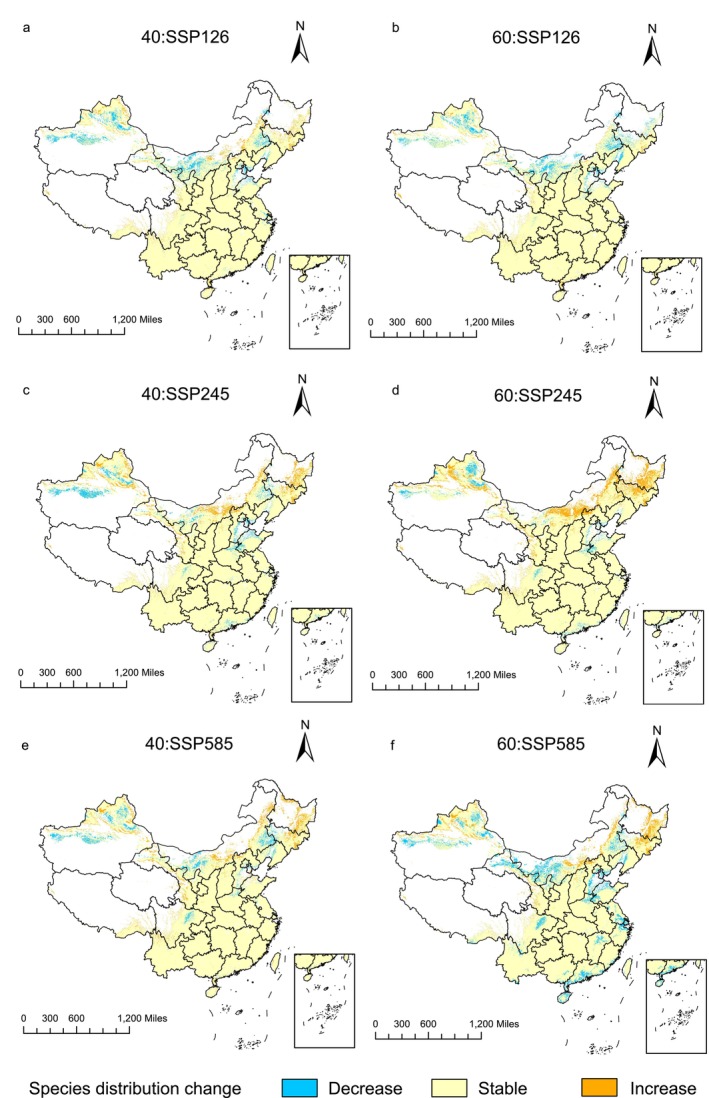
Spatial changes of geographical distribution of *B. chinensis* in SSP126 (a, b), SSP245 (c, d), SSP585 (e, f) scenarios in the 2041–2060s (a, c, e) and 2061–2080s (b, d, f).

In terms of changes in the suitable habitat area, compared to the current climate scenario (Table 3), the largest decrease in habitat area occurred under the SSP126 scenario for 2061–2080, with a reduction of 14.45 × 10^4^ km^2^. The largest increase in habitat area occurred under the SSP585 scenario for 2061–2080, with an increase of 40.63 × 10^4^ km^2^. Overall, the trend showed an increase in suitable habitat under the scenarios for 2041–2060, followed by a decrease in the scenarios for 2061–2080. As illustrated in the figures (Figure 7), the regions with the most significant decreases in suitable habitat were primarily concentrated in Xinjiang, Inner Mongolia, and Sichuan, while the regions with the largest increases were predominantly located in Gansu, Sichuan, Inner Mongolia, and Heilongjiang. In general, the suitable habitat for *B. chinensis* shifted with climate change, exhibiting similar distribution patterns under the same climate scenarios across different periods, but with varying degrees of change.

**TABLE 3:** Central particle size of curved Stem *B. chinensis* under different climatic scenarios across different time periods (×10^4^ km^2^).

Period	Climate scenario	Decrease	Stable	Increase	Decline rate (%)	Growth rate (%)
2041–2060	SSP126	21.32	414.12	17.14	4.89	3.94
	SSP245	17.06	418.48	30.97	3.92	7.11
	SSP585	17.26	418.28	30.13	3.96	6.92
2061–2080	SSP126	23.86	411.68	9.42	5.48	2.16
	SSP245	11.04	424.5	51.68	2.53	11.87
	SSP585	38.76	396.78	27.6	8.90	6.37

**FIGURE 8:**
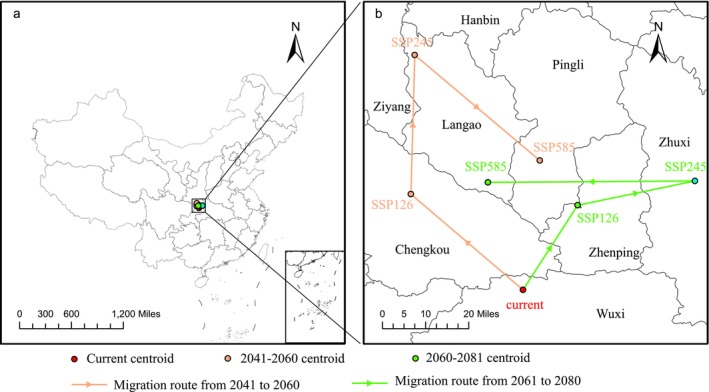
Geographical changes of centroid in *B. chinensis* under different climatic scenarios and periods [(b) is an enlargement of the part of (a)].

From the perspective of spatial patterns under different climate scenarios (Figure 8), the centroid of the suitable habitat for *B. chinensis* showed a northward migration. Currently, the centroid is located in Wuxi County, Chongqing (E109°7′41.6064″, N31°39′18.6156″). Under the SSP126 scenario for 2061–2080, the centroid migrated 37.65 km northeast to Zhenping County, Ankang, Shaanxi (E109°20′15.5256″, N31°56′34.062″), which represented the shortest migration distance. Under the SSP245 scenario for 2041–2060, the centroid migrated 96.72 km northwest to Langao County, Ankang, Shaanxi (E108°41′0.3012″, N32°26′16.6380″), representing the longest migration distance. These results indicated that, under future climate scenarios, rising temperatures due to global warming caused the suitable habitat for *B. chinensis* to shift northward, consistent with the general expectation that global warming would drive plant species to migrate toward higher latitudes.

### 3.5 The Main Environmental Factors Influencing the Distribution of *B. chinensis*

**FIGURE 9:**
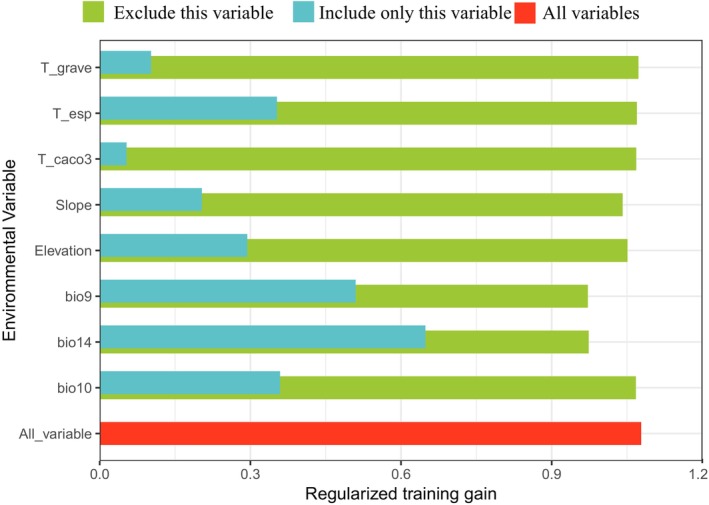
Regularized training gain of the MaxEnt model based on the jackknife test.

According to the MaxEnt model's predictions, four key environmental factors influenced the distribution of *B. chinensis* : precipitation of the driest month (bio14, 56.7%), slope (12.2%), mean temperature of the warmest quarter (bio10, 10.8%), and mean temperature of the driest quarter (bio9, 10.6%). The results from the jackknife test (Figure 9) showed that when only one factor was used, the highest regularized training gain was associated with bio14 (precipitation of the driest month), indicating that it contained the most information. This was followed by bio9 (mean temperature of the driest quarter) and bio10 (mean temperature of the warmest quarter), suggesting that these two factors had the greatest impact on the distribution of *B. chinensis* . When bio9 was excluded, the regularized training gain dropped the most, indicating that this factor contained the most unique information not shared by other variables. By combining the model's contribution rates with the jackknife test results, it was concluded that bio14 was the most important factor influencing the distribution of *B. chinensis* .

**FIGURE 10:**
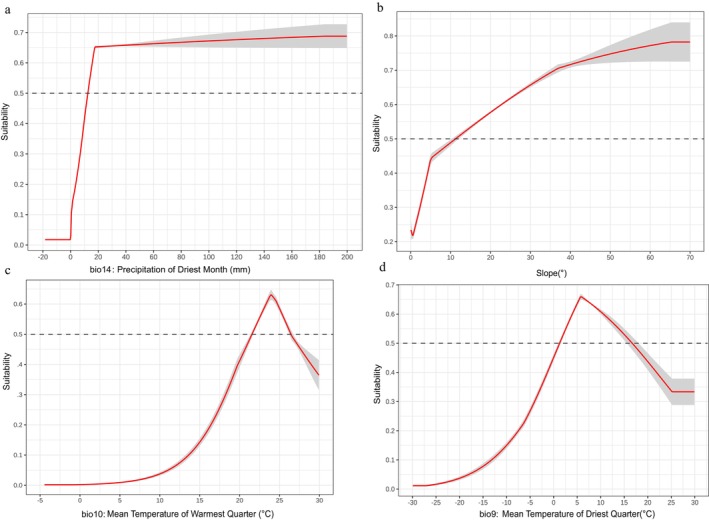
Single factor response curve of current climate [bio14 (a), Slop (b), bio10 (c), bio9 (d)].

For the five main environmental factors, the model generated response curves for each factor when used individually (Figure 10). A survival probability > 0.5 indicated that the corresponding range of values for that factor was suitable for plant growth. As shown in the figure, when the precipitation of the driest month (bio14) was less than 0 mm, the survival probability of *B. chinensis* was below 0.1. As precipitation increased, the survival probability also rose. When precipitation exceeded 12 mm, the survival probability became > 0.5. For the mean temperature of the driest quarter (bio10), when the temperature was below −13°C, the survival probability was < 0.1, but it increased as the temperature rose. The survival probability peaked at 6°C, after which it began to decline as the temperature exceeded 6°C. Thus, under current climate scenarios, the suitable ranges for these four environmental factors were as follows: precipitation of the driest month (bio14) > 12 mm, slope > 11°, average temperature in the warmest season (bio10) 22°C–27°C, and average temperature in the driest season (bio9) 2°C–17°C.

### 3.6 Multivariate Environmental Similarity Surface (MESS) and Most Dissimilar (MoD) Variable Analysis

**FIGURE 11:**
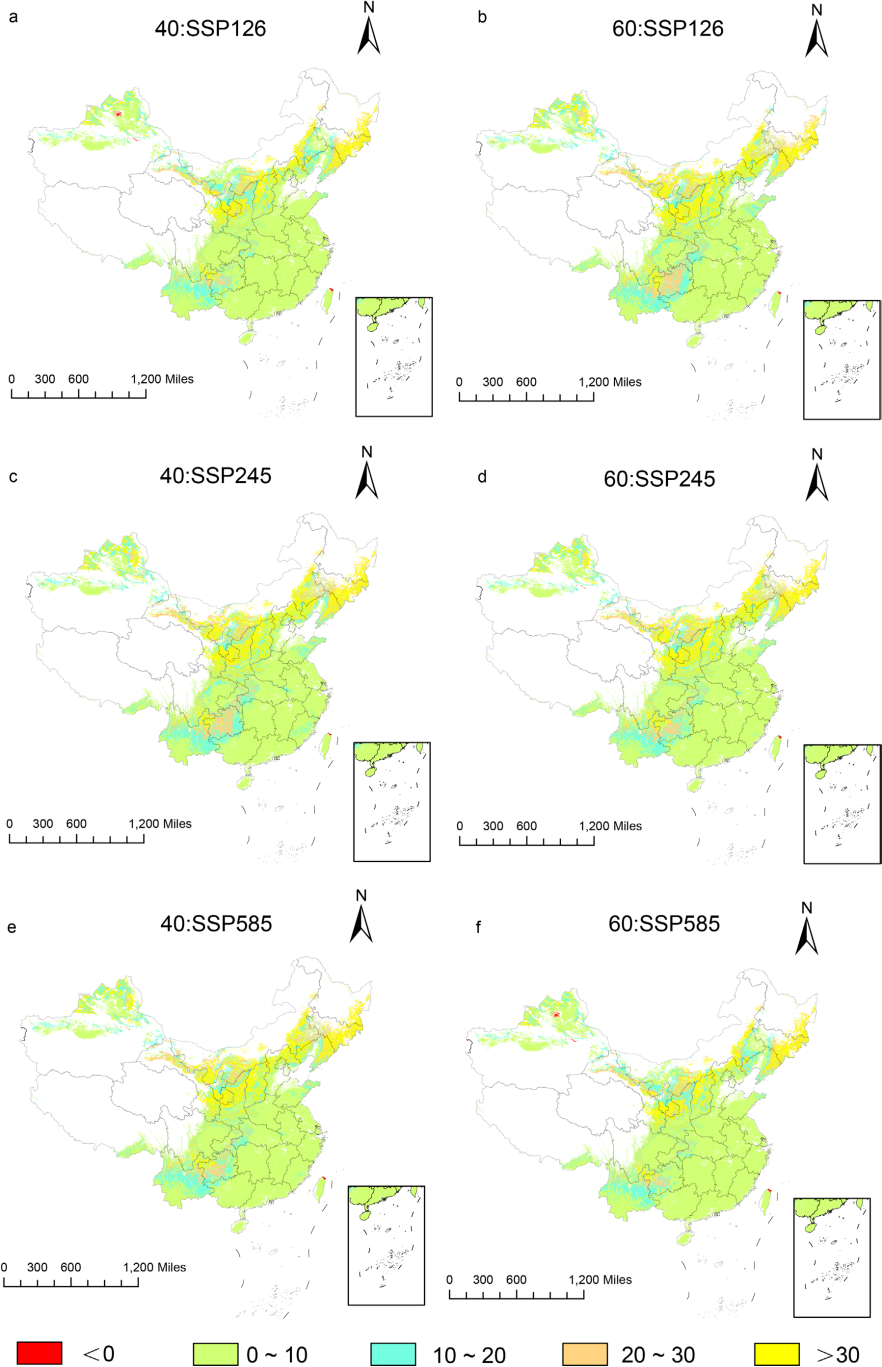
Multivariate environmental similarity surface of *B. chinensis* in SSP126 (a, b), SSP245 (c, d), SSP585 (e, f) scenarios in the 2041–2060s (a, c, e) and 2061–2080s (b, d, f).

**FIGURE 12:**
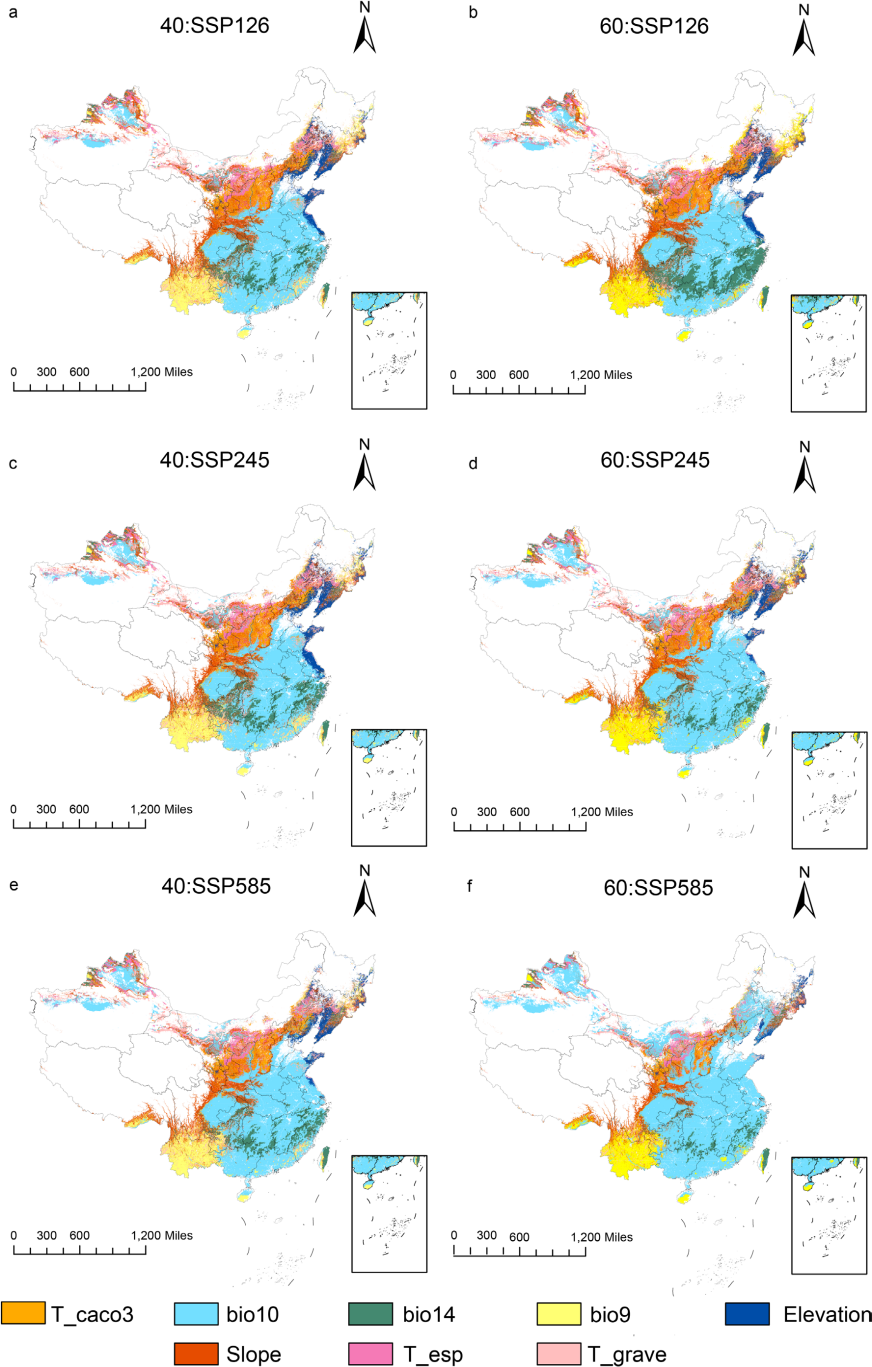
Most dissimilar variable analysis of *B. chinensis* in SSP126 (a, b), SSP245 (c, d), SSP585 (e, f) scenarios in the 2041–2060s (a, c, e) and 2061–2080s (b, d, f).

Compared to current climate scenarios, the mean Multivariate Environmental Similarity Surface (MESS) values for future climate scenarios ranged from 12.31 to 14.63, with all MESS values being positive and showing relatively small differences. This suggested that the differences between suitable habitats for *B. chinensis* in the future were minimal. The highest multivariate similarity occurred under the SSP245 scenario during the 2041–2060 period, indicating the lowest climate anomaly, while the lowest similarity occurred under the SSP585 scenario during the 2061–2080 period, indicating the highest climate anomaly (Figure 11). In addition, the SSP585 scenario consistently showed higher levels of anomaly compared to the other two scenarios. From a geographical perspective, MESS values were lower in southern regions than in northern regions, implying that climate anomalies were higher in the south. The analysis of the most dissimilar variables (Figure 12) showed that the main drivers of these changes were bio14 (precipitation of the driest month) and bio10 (mean temperature of the warmest quarter), with bio14 showing the most significant change, especially in the southern regions. Overall, climatic factors had the greatest influence, followed by topographic factors, with soil factors having the least influence.

### 3.7 Analysis of Niche Difference of *B. chinensis* in Different Periods and Different Climatic Scenarios

**FIGURE 13:**
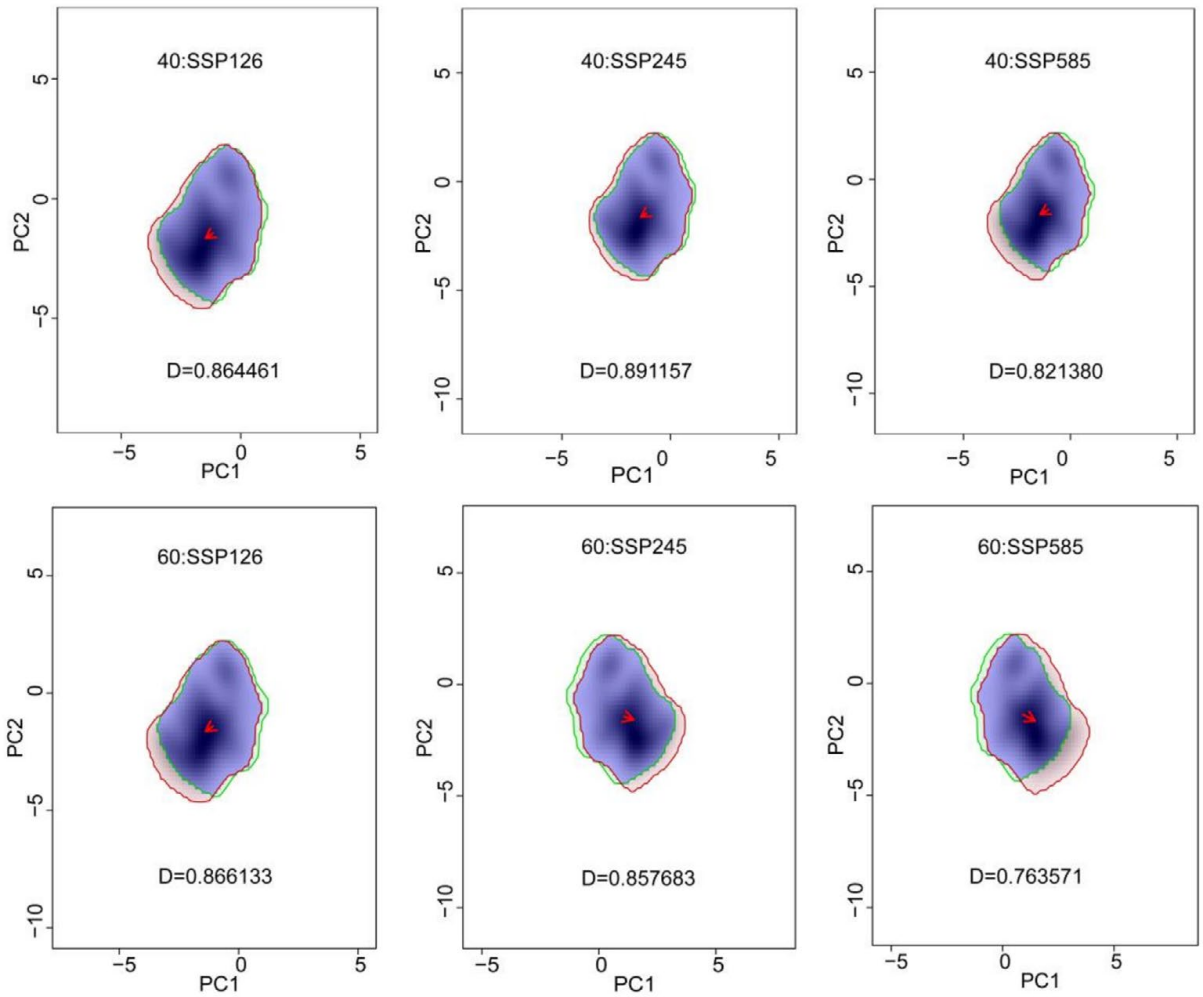
Niche differences of *B. chinensis* in different climatic scenarios in the future.

Using the Ecospat package in R, we visualized the ecological niche of *B. chinensis* under different climatic scenarios over various time periods. As shown in Figure 13, there was little niche divergence, with the highest niche overlap index (D) being 0.8912 and the lowest being 0.7634. Notably, with the exception of the SSP245 scenario during 2041–2060, which showed increased niche overlap, the overall trend demonstrated a progressive decline in niche overlap across temporal scales and varying climate scenarios. Furthermore, within the same model over different time periods, the niche overlap of *B. chinensis* under the SSP245 and SSP585 scenarios exhibited varying degrees of decline. Overall, this indicated that the common resources available to *B. chinensis* would decrease between the current and future periods. The ecological niche width of *B. chinensis* under different climatic scenarios and time periods was calculated using the ENMTools package. As shown in Table 4, B1 values ranged from 0.3746 to 0.4327, and B2 values ranged from 0.9534 to 0.9611. Except for the SSP245 scenario during the period of 2061–2080, under different scenarios during the same period, the B1 and B2 of *B. chinensis* increased with the enhancement of radiation intensity. The differences between B1 and B2 in each period were not significant, indicating that *B. chinensis* had wide ecological adaptability and tended to be a generalist species. Furthermore, compared to current climate scenarios, both B1 and B2 values increased in future climate scenarios, suggesting that *B. chinensis* would have access to more resources and exhibit greater environmental adaptability in the future.

**TABLE 4:** Ecological niche breadth of *B. chinensis* under different climatic scenarios and periods.

Period	Climate scenario	B1 (inverse concentration)	B2 (uncertainty)
Current		0.3746	0.9534
2041–2060	SSP126	0.3964	0.9564
	SSP245	0.4065	0.9578
	SSP585	0.4112	0.9587
2061–2080	SSP126	0.3804	0.9542
	SSP245	0.4327	0.9611
	SSP585	0.4111	0.9598

## 4 Discussion

### 4.1 The Utilization of MaxEnt Model

The MaxEnt model, based on the principle of maximum entropy, uses species occurrence points and environmental variables to simulate and predict potential distribution areas. Numerous studies have highlighted that the number of occurrence points and their spatial distribution significantly influence the accuracy of model predictions (Yao et al. 2023). To address this issue, we employed the ENMTools package in R to filter the occurrence points. This package randomly removes duplicate points within the same pixel, based on the resolution of the climate variables, ensuring that each pixel retains only a single occurrence point. In recent years, bioclimatic, topographic, and soil variables have become invaluable tools for studying biological invasions, predicting potential regional distributions, and assessing habitat suitability (Jiang et al. 2018; Jing et al. 2020; Wei et al. 2020; Zou et al. 2023). However, some studies have not adequately addressed the issue of redundant information introduced by highly correlated variables during the modeling process, which can negatively impact prediction accuracy (Duflot et al. 2018). In our study, we mitigated the effects of multicollinearity by using jackknife testing and Spearman correlation analysis, which enhanced the model's predictive accuracy.

Although the MaxEnt model has certain advantages over other species distribution models in terms of predictive accuracy, sample size requirements, and stability, it also has some limitations (Che 2022). Prior to importing environmental variables, it is necessary to standardize the resolution and boundaries of the data. The MaxEnt model predicts the maximum potential distribution of a species across a given region, though the actual distribution may not necessarily reach this theoretical maximum (Ta et al. 2021).

### 4.2 Environmental Factors Affecting the Distribution of *B. chinensis* With Curved Stems

Climate is widely recognized as the most significant environmental factor influencing species distribution (Jochum et al. 2007), primarily through two key aspects: temperature and moisture. Temperature provides the essential energy for plant life, while moisture is critical for supporting physiological processes (Fang 1991). However, the impact of these climate variables varies across species due to differences in their ecological characteristics. *B. chinensis* is highly adaptable, thriving in warm, sunny, and humid environments while exhibiting tolerance to both drought and cold conditions. It is not particularly demanding in terms of soil quality and can grow on a variety of land types, including both hilly and arid areas (He et al. 2023). MaxEnt model analysis reveals that the most influential environmental factors for the distribution of suitable habitats for *B. chinensis* are: precipitation of the driest month (bio14, 56.7%), slope (12.2%), mean temperature of the warmest quarter (bio10, 10.8%), and mean temperature of the driest quarter (bio9, 10.6%). Among these, precipitation in the driest month is the most critical factor, as adequate moisture is essential for the growth of this moisture‐loving species. Comparing these results with habitat predictions for other species, such as *Gentiana chinensis Kusnez*, for which the key factors include bio15, bio03, bio1, and clay content (Zou et al. 2023), and *Osmanthus fragrans* , which is primarily influenced by bio1 and bio10 (Yue et al. 2024), it is evident that different species exhibit distinct sensitivities to bioclimatic variables. While these variables play a fundamental role in plant growth, the specific sensitivities of each species must be assessed individually. Based on the analysis, the optimal habitat for *B. chinensis* under current climatic scenarios is characterized by the following: precipitation of the driest month > 12 mm, slope > 11°, average temperature of 22°C–27°C in the warmest season, and average temperature of 2°C–17°C in the driest season.

Although soil factors contribute relatively less to the overall distribution of *B. chinensis* , they still play an important role in its growth and habitat suitability. In our study, the environmental variables selected for analysis were limited to bioclimatic, soil, and topographic factors. However, it is important to note that species distributions are also influenced by biotic factors such as species interactions, dispersal limitations (Meier et al. 2012), evolutionary adaptations (Bush et al. 2016), and human activities (Qin et al. 2020). These factors, which were not included in the present study, can significantly affect the distribution patterns of *B. chinensis* . Thus, the variables considered in this analysis do not encompass all potential influences on the species' distribution.

### 4.3 Regional Distribution of Suitability of *B. chinensis*

In the context of global climate change, the persistent rise in temperatures may have detrimental effects on plant life (Xu and Xue 2013). These effects are likely to vary between species with broad versus narrow distributions. Plants with narrow distributions typically exhibit limited ecological adaptability, making them more susceptible to the impacts of climate change compared to species with broader distributions (Zhang et al. 2015). *B. chinensis* , with minimal environmental requirements, is widely distributed across most regions of China (Li, Shao, and Jiang 2022). Its primary production areas are currently located in southwestern, northwestern, and northeastern China (Ai 2023), confirming its broad geographic distribution. According to projections from the MaxEnt model, the current distribution of *B. chinensis* is largely concentrated in China's subtropical and temperate regions (encompassing both natural habitats and cultivated areas). The species occupies an estimated 435.5 × 10^4^ km^2^ of suitable habitat across several provinces, including Fujian, Hubei, Hunan, Guizhou, Henan, and Yunnan. The areas with the highest suitability for *B. chinensis* are primarily located in Hubei, Hunan, and Guizhou, which aligns with its known distribution patterns. While *B. chinensis* is a broadly distributed species, future climate scenarios predict a general reduction in its high‐suitability areas. Notably, under the SSP585 scenario for the period between 2061 and 2080, the model projects the largest decrease in suitable habitat. Some studies have shown that *B. chinensis* is capable of tolerating short‐term moderate to severe water stress (Li 2009). However, prolonged severe drought stress can cause irreversible damage to leaf cell membranes (Yang et al. 2018). Hence, we posit that beyond a certain threshold of climate warming, *B. chinensis* will experience significant adverse impacts on its survival and distribution.

The interaction between environmental suitability and shifts in geographic range is crucial in determining how species respond to climate change (Davis and Shaw 2001). In response to global climate change, many species are expected to migrate toward higher latitudes or altitudes (Root et al. 2003; Bertrand et al. 2011). However, the extent of these shifts may vary significantly among species, depending on their ecological characteristics and environmental tolerances. In comparison to current climate scenarios, the areas suitable for *B. chinensis* exhibit shifts across different time periods and climate change scenarios, showing consistent distribution trends but with varying degrees of change. Under the SSP126 scenario, suitable areas are projected to decrease overall. In contrast, the SSP245 scenario predicts an increase in suitable habitat, with the expansion primarily occurring toward higher latitudes and inland regions. In terms of spatial distribution, *B. chinensis* consistently shows a northward shift in its distribution centroid in response to climate change. The reduction in high‐suitability areas in the future could significantly impact existing production regions of *B. chinensis* . For example, traditional production areas may experience a shift or migration, suggesting that global climate warming will have a considerable influence on the geographic distribution of *B. chinensis* .

A multivariate environmental similarity surface (MESS) and most dissimilar variable analysis revealed a notable pattern: climate anomalies are significantly higher south of the Qinling‐Huaihe Line compared to northern regions. As climate warming intensifies, these anomalies become more pronounced, with northern regions also experiencing a gradual increase in climate anomalies. The primary drivers of these changes were identified as the precipitation of the driest month (bio14) and the mean temperature of the warmest quarter (bio10). Given the influence of these factors on the reduction of high‐suitability areas for *B. chinensis* , our analysis highlights the critical role of precipitation in shaping its distribution patterns.

In our study, we employed the natural breaks method, a technique developed within the Geographic Information System (GIS) software platform ArcGIS, to categorize areas suitable for the cultivation of *B. chinensis* . It is important to note, however, that different classification methods can yield varying results (Liu et al. 2013). For instance, the Intergovernmental Panel on Climate Change (IPCC) recommends a likelihood classification scheme with thresholds of 0~0.05~0.33~0.66~1 (Manning 2006). Alternatively, some approaches utilize two sets of sample points to establish thresholds by sorting the output values in ascending order within each set and selecting the points between the top 80% and bottom 20% as classification boundaries (Zhu et al. 2023). Additionally, artificial classification methods have also been applied (Yan et al. 2021). Therefore, it is crucial to define appropriate classification thresholds and assess their accuracy in relation to the actual geographic distribution of the species (Wang et al. 2020).

### 4.4 Ecological Niche Difference

Niche overlap refers to the degree to which two species share common resources or utilize multiple resources in tandem (Liu, Chen, and Zhang 2003). A wider ecological niche is typically associated with a more even species distribution and greater resource utilization capacity (Chen et al. 2014; Zhang et al. 2019). In this study, we observed that the ecological niche overlap of *B. chinensis* between the current and future periods remains relatively high. However, divergent climate scenarios demonstrate a marked decline in overall niche overlap, suggesting progressively constrained shared resources for *B. chinensis* under future conditions. The extent of niche overlap depends on both the ecological characteristics and the habitat of the species involved (Guo et al. 2019). It is important to note that this study did not account for the specific habitat conditions of *B. chinensis* , and as such, the findings may be subject to some degree of bias. The broad ecological niche of *B. chinensis* suggests that it is more likely to become a generalist species with strong environmental adaptability. Specifically, across different scenarios within the same period, both B1 and B2 values generally increased with higher radiative forcing, while no statistically significant differences were observed in these values between periods. This pattern can likely be attributed to the significant drought tolerance and cold hardiness of *B. chinensis* , coupled with its low soil requirements and high ecological adaptability (Chen 2024). Consequently, as future climate conditions become more favorable, the species may undergo range expansion into currently marginal areas.

## 5 Conclusion

The distribution of *B. chinensis* is primarily concentrated in the subtropical and temperate regions of China, spanning a broad geographic range that includes provinces such as Fujian, Zhejiang, Guangdong, Guangxi, Shaanxi, Sichuan, Guizhou, Hubei, Henan, and Yunnan (encompassing both natural habitats and cultivated areas). The species' distribution is primarily influenced by key environmental factors, including precipitation, slope, and temperature. Under future climate projections, the suitable habitat for *B. chinensis* shows varying trends, with an overall northward shift. As climate scenarios change, the availability of shared resources is expected to decline, while the resources that the species can utilize are predicted to increase. This shift suggests that *B. chinensis* will exhibit enhanced environmental adaptability in the future.

## Author Contributions

**Wei Lin:** conceptualization (equal), data curation (equal), software (equal), visualization (equal), writing – original draft (equal). **Feiran Hu:** supervision (equal), writing – review and editing (equal). **Guochun Fan:** software (equal), visualization (equal). **Qingxia Zhang:** data curation (equal), software (equal). **Min Deng:** software (equal), visualization (equal). **Xiangxin Xu:** visualization (equal). **Yibing Liu:** software (equal). **Junsheng Qi:** funding acquisition (equal), project administration (equal), resources (equal), supervision (equal).

## Conflicts of Interest

The authors declare no conflicts of interest.

## Supporting information


**Appendix S1:** ece372414‐sup‐0001‐AppendixS1.csv.

## Data Availability

The original contributions presented in the study are included in this paper, further inquiries can be directed to the corresponding author.
